# The Role of G-Protein-Coupled Receptor Proteolysis Site Cleavage of Polycystin-1 in Renal Physiology and Polycystic Kidney Disease

**DOI:** 10.3390/cells5010003

**Published:** 2016-01-21

**Authors:** Marie Trudel, Qin Yao, Feng Qian

**Affiliations:** 1 Molecular Genetics and Development, Institut de Recherches Cliniques de Montreal, Universite de Montreal, Faculte de Medecine, Montréal, Québec H2W 1R7, Canada; marie.trudel@ircm.qc.ca; 2 Department of Medicine, Division of Nephrology, University of Maryland School of Medicine, Baltimore, MD 21201, USA; qyao@medicine.umaryland.edu

**Keywords:** polycystin, polycystic kidney disease, GAIN domain, GPS motif, cis-autoproteolysis, adhesion GPCR

## Abstract

Polycystin-1 (PC1) plays an essential role in renal tubular morphogenesis, and PC1 dysfunction causes human autosomal dominant polycystic kidney disease. A fundamental characteristic of PC1 is post-translational modification via cleavage at the juxtamembrane GPCR proteolysis site (GPS) motif that is part of the larger GAIN domain. Given the considerable biochemical complexity of PC1 molecules generated *in vivo* by this process, GPS cleavage has several profound implications on the intracellular trafficking and localization in association with their particular function. The critical nature of GPS cleavage is further emphasized by the increasing numbers of *PKD1* mutations that significantly affect this cleavage process. The GAIN domain with the GPS motif therefore represents the key structural element with fundamental importance for PC1 and might be polycystic kidney disease’s (PKD) Achilles’ heel in a large spectrum of *PKD1* missense mutations. We highlight the central roles of PC1 cleavage for the regulation of its biogenesis, intracellular trafficking and function, as well as its significance in polycystic kidney disease.

## 1. Introduction

Autosomal dominant polycystic kidney disease (ADPKD) is the most common monogenic disorder affecting one in 500–1000 individuals worldwide [1]. It is caused by mutations in either *PKD1* (85%–90%) [2] or *PKD2* (10%–15%) [3], which encodes polycystin (PC1) [4] or polycystin-2 (PC2), respectively. ADPKD is characterized by the formation of kidney cysts that gradually replace normal kidney parenchyma [5,6]. This process can initiate early in development [7,8] and continues throughout lifetime, leading to kidney failure usually after the fifth decade of life [9]. 

**Figure 1:**
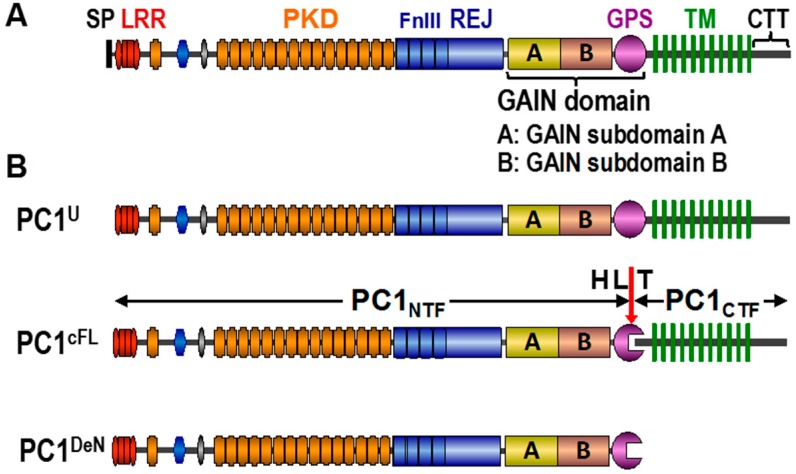
A schematic diagram of the domain organization of polycystin-1 and various products generated by cleavage at the GPCR proteolysis site (GPS) motif within the GAIN domain. (**A**) Schematic diagram of the structure of polycystin-1. SP, signal peptide; LRR, leucine-rich repeat; PKD, polycystic kidney disease (PKD) domains; REJ, receptor for egg jelly module with its four fibronectin III domains (FnIII); GAIN, GPCR autoproteolysis-inducing domain; A, GAIN subdomain A; B, GAIN subdomain B; GPS, G-protein-coupled receptor proteolytic site motif; TM, transmembrane domain; CTT, C-terminal tail. (**B**) Polycystin-1 products generated by GPS cleavage. PC1^U^, uncleaved full-length PC1; PC1^cFL^, cleaved full-length PC1 in which the two cleavage fragments, PC1_NTF_ and PC1_CTF_, remain non-covalently associated within the GAIN; the HL*T tripeptide within the GPS motif is indicated; PC1^deN^, a separate form of the PC1_NTF_ molecule derived from PC1^cFL^ once it has dissociated from PC1_CTF_.

PC1 is a 4302-amino acid (aa) 11-transmembrane (TM) receptor-like glycoprotein with a large N-terminal extracellular region of 3072 aa and a short cytoplasmic C-terminal tail (CTT) of ~200 aa [4] (Figure 1A). The N-terminal extracellular region contains a set of domains involved in protein-protein interactions and the ~1000 aa receptor for egg jelly (REJ) module that harbors four FnIII domains [10,11]. Situated at the base of the extracellular region is the 50-aa GPCR proteolysis site (GPS) motif [12,13]. The GPS motif was first identified in a neuronal GPCR, CIRL/latrophilin [14], and has recently been recognized as a part of the larger GPCR autoproteolysis-inducing (GAIN) domain that is also present in PC1 [15]. The GAIN domain is a defining feature of the adhesion GPCRs (aGPCRs), the second largest subgroup of GPCRs in the human genome [16,17]. The CTT of PC1 is responsible for regulating a number of intracellular signaling pathways, including Ca^2+^ [18,19], Wnt [20], mTOR [21] and energy metabolism [22,23]. The CTT fragment can bind heterotrimeric G-proteins *in vitro* [24] and mediate AP activation via heterotrimeric G proteins, suggesting that PC1 could be an atypical GPCR [25]. CTT of PC1 can be released by γ-secretase-mediated cleavage and regulates the CHOP pathway by binding the transcription factors TCF and CHOP, disrupting their interaction with the common transcriptional co-activator p300 [26,27]. CTT contains a coiled-coil domain that binds PC2 [28,29,30], resulting presumably in the formation of a receptor-channel complex at the plasma membrane, as well as the primary cilium [31], an organelle that is most relevant to the pathogenesis of ADPKD [32,33,34]. The ciliary PC1/2 complex is proposed to mediate signaling pathways in response to mechanical or chemical signals, although the underlying mechanism remains unclear [31,35].

A fundamental property of PC1 is post-translational modification by proteolytic cleavage at the juxtamembrane GPS motif [13,36]. We have reported that PC1 undergoes cleavage at the HL*T^3041^ tripeptide sequence (*: scissile bond, with amino acid numbering based on mouse PC1) within the GPS shortly after synthesis in the ER, resulting in two fragments, PC1_NTF_ and PC1_CTF_ [37]. GPS cleavage of PC1 occurs via a *cis*-autoproteolytic mechanism without requiring exogenous proteases with similar parameters as in the aGPCR EMR2 in which the mechanism was first discovered [38]. A unique outcome of the cleavage is that the two fragments remain tightly and non-covalently associated to form a heterodimeric molecule termed PC1^cFL^ [39]. GPS cleavage plays a critical role for PC1 function *in vivo* as demonstrated by the generation and characterization of the *Pkd1*^V/V^ knockin mouse that express a non-cleavable form of PC1 termed PC1^V^ [40]. The *Pkd1*^V/V^ is the first engineered *Pkd1* mouse model with a missense mutation, which replaces the critical threonine residue at amino acid position 3041 to a valine at the HL*T^3041^ and thereby blocks GPS cleavage of PC1. The critical nature of GPS cleavage is further highlighted by the effect of the increasing number of *PKD1* mutations that significantly alter this process. This review will discuss the fundamental property of PC1 cleavage for regulating its own biogenesis, intracellular trafficking and function, as well as its significance in polycystic kidney disease. 

## 2. Specific Role of GPS Cleavage of Polycystin-1 in the Kidney 

### 2.1. Potential Role of PC1^U^ in Kidney Development and Proximal Nephron Segments

Expression of PC1 in mouse kidneys is developmentally regulated [39,41]. Consistent with the highest levels during early renal organogenesis, PC1 is highly expressed in the embryonic kidneys. The expression level in kidneys decreases through postnatal Days P3–14 and becomes barely detectable at P21 and adult in comparison to most other organs [39]. Remarkably, PC1 also has a development-specific cleavage pattern in the kidney [41]. The proportion of PC1^U^ is maximal at e13.5 (~50%) and decreases gradually during successive embryonic stages with ~20% at e15.5 and very little at birth. The gradual decrease is accompanied by the gradual increase in the proportion of PC1^cFL^, which becomes the predominant form of PC1 in distal nephrons (>90%) as in most adult tissues. However, a significant portion of PC1 remains as the PC1^U^ (~50%) form in proximal tubular cells even after birth [40]. Therefore, GPS cleavage of PC1 in the kidney appears to be developmentally and nephron segment-specifically regulated. 

Analyses of the *Pkd1*^V/V^ mouse model in comparison to the *Pkd1* null mice demonstrate a critical role of GPS cleavage for the full biological function of PC1 *in vivo* [40]. The *Pkd1*^V/V^ mutant mice express non-cleavable PC1^V^ at a level that is comparable to that of PC1^U^ and lack GPS-cleaved PC1 molecules. The level of PC1^V^ expressed is therefore as low as 10%–20% of the total PC1 amount (*i.e*., PC1^U^ plus PC1^cFL^) in wild-type controls. The *Pkd1*^V/V^ animals are viable with virtually normal appearing kidneys at birth [40], whereas *Pkd1* null mice develop very severe cystic kidneys starting at e15.5 and are embryonically lethal [42,43,44]. One explanation for the hypomorphic nature of the *Pkd1^V^* allele would be that the resulting PC1^V^ has a partial, but otherwise redundant function of PC1^cFL^ to allow the animals to get through embryonic development, but is insufficient to prevent cystogenesis in the postnatal period. However, the *Pkd1^L3/L3^* mutant mice that express ~20% of normal PC1 levels exhibit cysts starting from e15, and cysts progress gradually; half of these die at 1–2 months, while others survive several months [45]. In this case, uncleaved and cleaved PC1 proteins are reduced presumably in equal proportions. The pattern of rescue in *Pkd1*^V/V^ kidneys correlates with the high proportion of PC1^U^ expressed in early embryonic kidneys and proximal tubules in wild-type mice [40,41]. These data together imply that the uncleaved PC1 form is more active or efficient than the cleaved PC1 during development, and this function is rescued by PC1^V^ to prevent embryonic cyst formation in the *Pkd1*^V/V^ kidneys. PC1^V^ likely resembles PC1^U^, not only structurally, but also functionally. These considerations support the notion that PC1^U^ may play a key role in embryonic kidney development for proper renal epithelial tubular differentiation and maturation. 

One possibility would be that PC1^U^ is critical in the process of the tubular convergent extension in embryonic kidneys, which is defective in the *Pkd1* null mice [41]. Based on ablation of cilia by the early inactivation of ciliary protein Kif3a [46] or Ift20 [47] that cause cysts starting only from postnatal Day 5 and not during renal embryonic or fetal development, the function of PC1^U^ is likely ciliary independent. Similarly, PC1^U^ may play a key role in maintaining the intact structure and homeostasis of proximal nephron segments. It is conceivable that PC1^U^ can assume a specific function during early nephrogenesis and in the normal proximal tubules via interactions with unknown ligand or other molecules at the plasma membrane. 

### 2.2. Essential Role of PC1^cFL^ for Distal Nephron Segments in the Postnatal Period 

*Pkd1*^V/V^ mice develop rapid renal cysts starting at P3 in distal nephron segments, culminating in death by ~3 weeks with renal failure [40]. These mutants have a more rapid course of cystogenesis and a shorter life span compared to other hypomorphic mouse models with reduced *Pkd1* expression. For example, the *Pkd1^L3/L3^* mice have a much slower rate of cystic progression and can survive several months [45]. Similarly, the *Pkd1^nl/nl^* mice with 13%–20% of normal *Pkd1* transcript display distal renal cysts and died at 1–2 months of age or even later [48]. Taken together, these data indicate that PC1^V^ has little function in distal nephron segments after birth, in which PC1 is present predominantly in cleaved form in wild-type mice. These considerations support the notion that GPS cleavage of PC1 is essential in kidneys for the homeostasis of distal nephron segments in the postnatal period and that PC1^cFL^ plays a key function for maintaining intact distal nephron morphology at the postnatal maturation stage. 

## 3. Structural Basis of GPS Cleavage and the Heterodimeric Association 

**Figure 2:**
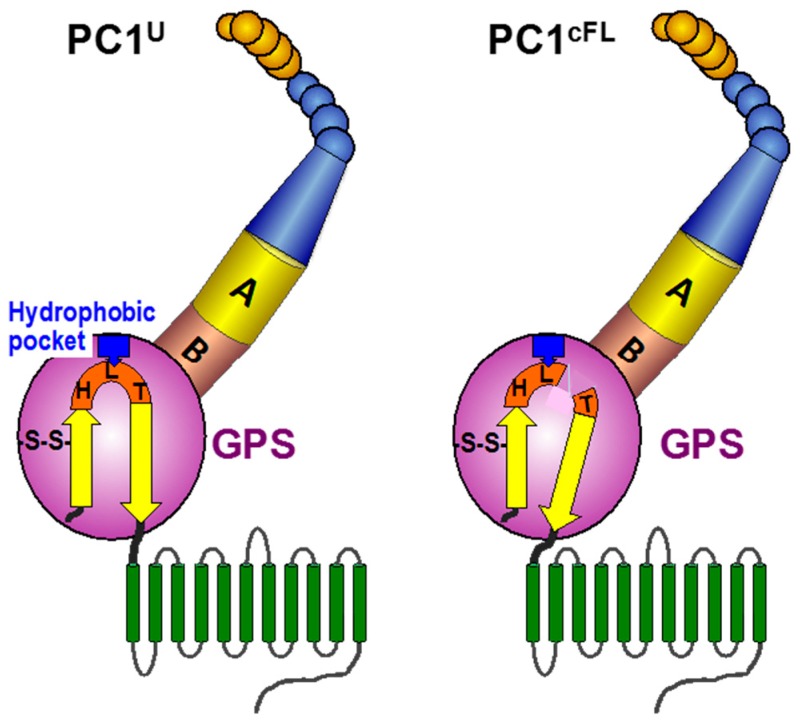
Schematic depiction of the GAIN domain in uncleaved (PC1^U^) and cleaved heterodimeric (PC1^cFL^) forms of polycystin-1. The model is based on an analogy with the structure of the GAIN domains of the adhesion GPCRs. A, GAIN subdomain A; B, GAIN subdomain B. In PC1^cFL^, the tight association of the last β-strand harnesses the cleavage-released force to generate a small tilt. The disulfide bridge (-S-S-) at the penultimate β-strand is shown. The trapping of the Leu (L) side chain of the HLT tripeptide within a conserved hydrophobic pocket is depicted by a blue box. The color code of the structural elements is identical as in Figure 1.

The function of the cleaved form of the PC1 molecule may be dependent on its heterodimeric composition [13,39]. One interesting clue comes from the parallel studies of aGPCRs, which contain a GPS motif at the juxtamembrane position and a large number of extracellular domains as PC1, although their actual domains and folds differ from the latter [16,17]. A recent crystallographic study of aGPCRs by Arac *et al*. [15] provided critical insights into both the structural basis of GPS cleavage and the association of cleaved subunits, which have important implications for PC1. The GPS motif forms five β-strands that are tightly integrated into the ~320-residue GPCR autoproteolysis-inducing (GAIN) domain. In the structure of the uncleaved GAIN domain, the HL*T tripeptide of the GPS motif is positioned at a sharply kinked loop between the last two β-strands. This distorted and strained geometry drives the equilibrium toward an N–O shift to facilitate ester formation and provides the necessary driving force for *cis*-autoproteolysis. Three structural elements are responsible for keeping the sharp β-turn in place: (1) two disulfide bonds between neighboring β-strands; (2) an extensive network of hydrophobic interactions between the last β-strand and other residues within the GPS motif; and (3) the trapping of the Leu (L) side chain of the HL*T within a conserved hydrophobic pocket. Significantly, the cis-autoproteolytic cleavage relaxes the sharp kink and results in a small tilt in the last β-strand. This small tilt is thought to be required for activation of the aGPCRs [15,49]. Deletion of the NTF (equivalent to PC1_NTF_) resulted in constitutive activation for some aGPCRs [50,51], suggesting that NTF association might normally prevent constitutive activation. This finding has led to the suggestion that such cleavage and association may allow regulation of aGPCR activity [15,49,50,52]. PC1 shares most of these structural elements, albeit it has only one disulfide bond, C3015-C3043, as depicted in Figure 2. 

## 4. Role of GPS Cleavage for PC1 Trafficking 

Recent works indicate that GPS cleavage also plays a critical role in intracellular trafficking of PC1 [39]. PC1^U^ resides primarily in the ER as demonstrated by *N*-glycosylation analysis. PC1^U^ does not seem to exit the ER or to reach the *cis*-Golgi network at the steady state. Critical insights into the biochemical status of the PC1^U^ form were provided by the analysis of PC1^V^ in *Pkd1*^V/V^ mice [39,40]. We found that this non-cleavable PC1 form can exit the ER and transit through the Golgi. It is quite likely that PC1^U^ has equivalent trafficking properties, although these cannot be directly detected by the *N*-glycosylation analysis. A simple explanation is that the PC1^U^ molecules can exit the ER, but at a much slower rate than the PC1^cFL^ molecules [39]. Consequently, the rate of GPS cleavage that converts PC1^U^ to PC1^cFL^ determines ER-to-Golgi transition of PC1, as well as the relative distribution of the two PC1 forms *in vivo.* The PC1^U^ trafficking *in vivo* is thus probably affected by the rate of GPS cleavage that decreases the pool of PC1^U^ and concomitantly increases the PC1^cFL^ population. These considerations support the notion that the rate of cleavage determines the rate of trafficking of PC1 and, therefore, the amount of functional PC1 at the cellular sites at which PC1 functions. Taken together, the native PC1^U^ has the potential to transit independently of the GPS cleavage, although the renal cell physiology promotes PC1 GPS cleavage prior to trafficking from the ER to the Golgi [39].

The GPS cleavage plays a unique role in regulating PC1 trafficking to primary cilia in the form of the endogenous PC1/2 complex via a two-step mechanism [53]. The experimental evidence suggested a sequence of steps for ciliary trafficking of PC1. First, PC1 requires physical interaction with PC2 via their cytoplasmic C-termini to exit from the ER, whereby the resulting PC1/2 complex binds Rabep1. Cleavage is necessary to enable PC1/2 to traffic to Golgi. Second, once arriving at the trans-Golgi, the PC1/2-bound Rabep1 at the TGN recruits GGA1 [54] and the small GTPase Arl3 [55] sequentially to enable subsequent ciliary targeting.

Recently, we have found that PC1_NTF_ can be detached from the PC1^cFL^ molecule and becomes an independent form of PC1, termed PC1^deN^ (Figure 1B) [39]. Native PC1^deN^ localizes at the plasma membrane of renal epithelial cells. PC1^deN^ was initially thought to traffic autonomously to reach the plasma membrane and play a critical functional role in renal homeostasis. However, evidence showed that the trafficking of PC1^deN^ does not occur autonomously, but occurs by virtue of association with PC1_CTF_ in the form of PC1^cFL^, followed by subsequent subunit dissociation. The biochemical analyses have indicated the presence of at least two regions within the PC1_CTF_ subunit, one within the first transmembrane domain and the other in the C-terminal tail, for PC1^deN^ trafficking. The function of PC1^deN^ remains to be determined.

## 5. Significance of Individual Mutations at the Critical HL*T Tripeptide for PC1 Trafficking and Function

A recent study has used *Pkd1^L3040H^-BAC* transgenic lines to analyze the functional role of GPS cleavage *in vivo* [56]. This transgene contains a single amino acid substitution L3040H at the penultimate position of the HL*T tripeptide cleavage site and expresses a non-cleavable PC1-L3040H protein. Although the result of the study also supports a critical role of GPS cleavage for PC1 function, it has an important difference to that with the *Pkd1*^V/V^ knockin mice. In contrast to the *Pkd1*^V/V^ mice with an apparent normal phenotype at birth, none of the three independent *Pkd1^L3040H^-BAC* transgenic lines rescued the embryonic lethality of *Pkd1*^−/−^mice, indicating a complete loss-of-function of the PC1-L3040H mutant. The three *Pkd1^L3040H^-BAC* transgenic lines expressed very different levels of the mutant protein. The distinctive outcomes between PC1-L3040H and PC1^V^ are unlikely due to different expression levels or other factors based on methodological difference: transgenic *vs.* knockin.

Significantly, there is a key biochemical difference between the two non-cleavable mutants: PC1^V^ consistently acquires EndoH resistance [39,53], whereas PC1-L3040H remains completely EndoH sensitive [56]. These data indicate that PC1^V^ can traffic to the Golgi compartment as the endogenous PC1 molecules, while PC1-L3040H is retained in the ER and defective in trafficking. The different properties of the two mutants most likely result from the position and nature of the amino acid substitution. The Leu3040 residue is highly conserved in all GPS motifs within the GAIN domains and forms the hydrophobic pocket at the sharp kink of the scissile bond based on the structural analysis of the aGPCR GAIN domains [15]. Substitution with the bulky and charged His at this position in PC1-L3040H may therefore critically alter the conformation of the GAIN domain and thereby secondarily confine PC1-L3040H to the ER and disrupts exit from the ER. In summary, bulky amino acid substitutions at position 3040 may lead to the destruction of the GAIN domain conformation, rather than the mere disablement of GPS proteolysis. Surely, if the GAIN domain unfolds, no GPS cleavage can take place, as indicated by Arac *et al.* [15] (Figures 5D and S4 of the reference). Taken together, loss-of-function observed in the PC1-L3040H mutant is likely a secondary consequence of the unfolding of the GAIN domain or global structural disruption, making it difficult to attribute its outcome exclusively to the inhibition of GPS cleavage. 

In the PC1^V^ mutant, on the other hand, the nucleophile threonine at position 3041 is replaced by valine, which differs from the former solely by one functional group at the very terminus of the side chain (-OH *vs.* -CH3) [37,40]. While effectively preventing cleavage by blocking the nucleophile attack, the critical initial step of *cis*-autoproteolysis [37], this smallest possible change seems less likely to significantly alter the conformation surrounding the cleavage site, as experimentally supported by its ability of acquiring EndoH resistance [39,53]. The equivalent mutation did not cause conformational changes in the cis-autoproteolytic protein, as shown by structural analysis [57]. In addition, it is fully consistent with the finding of Arac *et al.* [15] that GPS cleavage *per se* is not required for trafficking of the GAIN domain-containing proteins. Taken together, the T3041V mutation in PC1^V^ blocks cleavage without major “side effects”, thereby providing pertinent information about a critical and restricted role of GPS cleavage for PC1 function *in vivo*.

In summary, the findings with the two PC1 mutants reveal the importance and specific roles of individual residues within the sharply-kinked HL*T tripeptide of the GAIN domain. Furthermore, the data highlight the importance of structural modification associated with specific mutations, biochemical characterization of PC1 mutation on GPS cleavage and trafficking for assessing the function of GPS cleavage. These considerations have important implications toward the design of appropriate therapy, as discussed below.

## 6. Defective GPS Cleavage of PC1 in Polycystic Kidney Disease 

All of the disease-associated missense mutations located in the GAIN domain and the adjacent REJ module of PC1 (Figure 1) analyzed so far impaired or disrupted cleavage [13,15,56,58,59]. The GAIN-REJ region has been determined to be critical for effective GPS cleavage of PC1 [13]. *PKD1* mutations are distributed across the entire gene. However, approximately 30% of all pathogenic *PKD1* missense and small deletion/insertion mutations are located in the REJ-GAIN region (http://pkdb.mayo.edu/) and have the potential to affect PC1 cleavage. Mutations within PC1_CTF_ can also affect cleavage in humans and mice [39]. Recent studies have shown that incompletely penetrant *PKD1* alleles can impair PC1 cleavage [59]. Collectively, defective cleavage of PC1 plays an important role in the pathomechanism of PKD. 

Much of the ongoing effort toward therapeutic strategies has been directed at targeting dysregulated pathways associated with PKD [60,61,62]. This paradigm has been met with limited success, partly because of the uncertainty of how any of these pathways are linked to cyst formation [63,64]. A more effective and powerful strategy would be to target the mutant PC1 itself, the primary cause of PKD. The GAIN domain and the adjacent REJ module might be PKD’s Achilles' heel in a significant number of patients with *PKD1* missense and in-frame small deletion/insertion mutations. Given the critical importance of GPS cleavage for PC1 function, GPS cleavage might be an excellent target for the PC1-directed therapy, which would be amenable for this class of *PKD1* mutations. One approach may be the use of chemical chaperones or other small molecules to overcome or bypass impaired GPS cleavage of these PC1 mutants, analogous to the concept shown for the vasopressin receptor and CFTR [65,66]. A similar strategy was successfully applied for the development of the new transformational drugs for cystic fibrosis [67].
